# The use of transcutaneous CO_2_ monitoring in cardiac arrest patients: a feasibility study

**DOI:** 10.1186/s13049-014-0070-2

**Published:** 2014-11-29

**Authors:** Sung-Hyuk Choi, Jung-Youn Kim, Young-Hoon Yoon, Sung-Jun Park, Sung-Woo Moon, Young-Duck Cho

**Affiliations:** Department of Emergency Medicine, Korea University College of Medicine, 73, Inchon-ro, Sungbuk-gu, Seoul South Korea

**Keywords:** Return of spontaneous circulation, Transcutaneous carbon dioxide, Blood gas monitoring, Cardiac arrest

## Abstract

**Background:**

Prediction of the return of spontaneous circulation (ROSC) in cardiac arrest patients is a parameter for deciding when to stop cardiopulmonary resuscitation (CPR) or to start extracorporeal CPR. We investigated the change in transcutaneous PCO_2_ (PtcCO_2_) in cardiac arrest patients.

**Methods:**

This study was carried out as a retrospective chart review. Patients with out-of-hospital cardiac arrest or in-hospital cardiac arrest within the emergency department were included. PtcCO_2_ monitoring with a V-Sign™ combined monitor (SenTec Inc., Therwil, Switzerland) was applied to patients at the start of CPR. We divided the included patients into the ROSC group and the no ROSC group. The ROSC group was subdivided into those achieving ROSC <15 min CPR and >15 min CPR. The change in the PtcCO_2_ value was analyzed at 0 min, 5 min, 10 min, and 15 min from PtcCO_2_ stabilization and was compared among the groups.

**Results:**

A total of 42 patients were enrolled. Twenty-eight patients achieved ROSC; 13 patients achieved ROSC <15 min CPR and 15 patients achieved ROSC >15 min CPR. Fourteen patients expired without ROSC. The absolute values of PtcCO_2_ was lower in the ROSC group than in the no ROCS group. The PtcCO_2_ change over time had a tendency to decrease or to remain constant in the ROSC groups. In contrast, all patients in the no ROSC group experienced an increase in the PtcCO_2_ change during CPR except one case.

**Conclusions:**

PtcCO_2_ monitoring provides non-invasive, continuous, and useful monitoring in cardiac arrest patients.

## Background

The return of spontaneous circulation (ROSC) in cardiac arrest patients is meaningful for several reasons. It can help in deciding when to stop cardiopulmonary resuscitation (CPR). It also provides a parameter that can help decide when to start extracorporeal CPR (ECPR) despite the fact that the indication for CPR varies across critical care settings [1]. Many factors such as etiology, witnessed or not, location, initial electrocardiogram (ECG), bystander CPR, and time to hospital, are considered to predict ROSC. End-tidal CO_2_ (etCO_2_) monitoring has been known, heretofore, as a useful tool to predict the ROSC of a cardiac arrest patient [2]. However, several factors affect etCO_2_, such as airway obstruction, low cardiac output, and pulmonary edema, which are frequently found in cardiac arrest patients [3].

Transcutaneous PCO_2_ (PtcCO_2_) monitoring was introduced about 30 years ago. With advances in technology, the use of PtcCO_2_ monitoring has been expanded to adult patients [4-7]. In several clinical situations, a PtcCO_2_ analysis is preferred to an etCO_2_ analysis [8-10].

We observed that PtcCO_2_ monitoring is easily applied to and gives continuous information on gas changes in critically ill patients. Some investigators have reported that PtcCO_2_ shows good agreement with PaCO_2_ under the condition of adequate cardiac output, but it becomes flow-dependent during a low-flow shock in a laboratory, intensive care unit, or operating room [11,12]. On the basis of these findings, in this study, we investigated the change of PtcCO_2_ in cardiac arrest patients.

## Materials and methods

This study was carried out as a retrospective chart review and approved by the institutional review board of Korea University Guro Hospital. The study was conducted in Korea University Guro Hospital Emergency Department (ED) from March 2013 to December 2013. Patients with out-of-hospital cardiac arrest (OHCA) or in-hospital cardiac arrest (IHCA) within the ED were included. We excluded cases for whom transcutaneous monitoring was not applied or that failed to provide sufficient quality of PtcCO_2_ monitoring data. We also excluded patients under the age of 18 years. We divided the included patients into ROSC and no ROSC. The ROSC group was again divided into the ROSC in less than 15 min of CPR and the ROSC in more than 15 min of CPR. In this study, ROSC was defined as a case in which the evidence of a palpable pulse or a measurable blood pressure was sustained for >20 min.

The PtcCO_2_ monitoring device was a V-Sign™ combined monitor (SenTec Inc., Therwil, Switzerland). The V-Sign™ combined monitor is composed of display device and sensor using a heated electrode membrane. When a sensor is attached to the patient’s earlobe, it is heated up to 42°C. As the heated membrane vasodilates the skin capillaries, PtcCO_2_ comes in agreement with arterial PCO_2_.The waveform of PtcCO_2_ is displayed on a monitor, and it stabilizes in about 5 min after attachment of the sensor.

Two investigators who were blinded to the study objectives reviewed the patient charts. The collected data were gender, age, location of cardiac arrest, bystander CPR, OHCA versus IHCA, duration of CPR in the hospital, epinephrine used in the first 15 min of in-hospital CPR, time from the EMS call to hospital arrival, sodium bicarbonate use, and initial PtcCO_2_. The change in PtcCO_2_ during CPR was investigated in the ROSC <15 minute CPR group, the ROSC >15 minute CPR group, and the no ROSC group. The PtcCO_2_ value was analyzed at 0 min, 5 min, 10 min, and 15 min from the PtcCO_2_ stabilization and was compared in three groups. When the two chart reviewers reported conflicting data for continuous variables, the mean value was used in the continuous variables. For categorical variables, a third investigator reviewed the chart and determined which data were to be used.

For statistics, the Kruskal-Wallis test was used to compare the mean value of the continuous variables, and the chi-squared analysis was used for categorical variables with the SPSS 17.0 software package (IBM, Chicago, IL, USA). Repeated measures ANOVA with a post hoc test was used to test for the significance of the change in PtcCO_2_ value with time. We considered a *p* value of less than 0.05 to be statistically significant.

## Results

**Figure 1:**
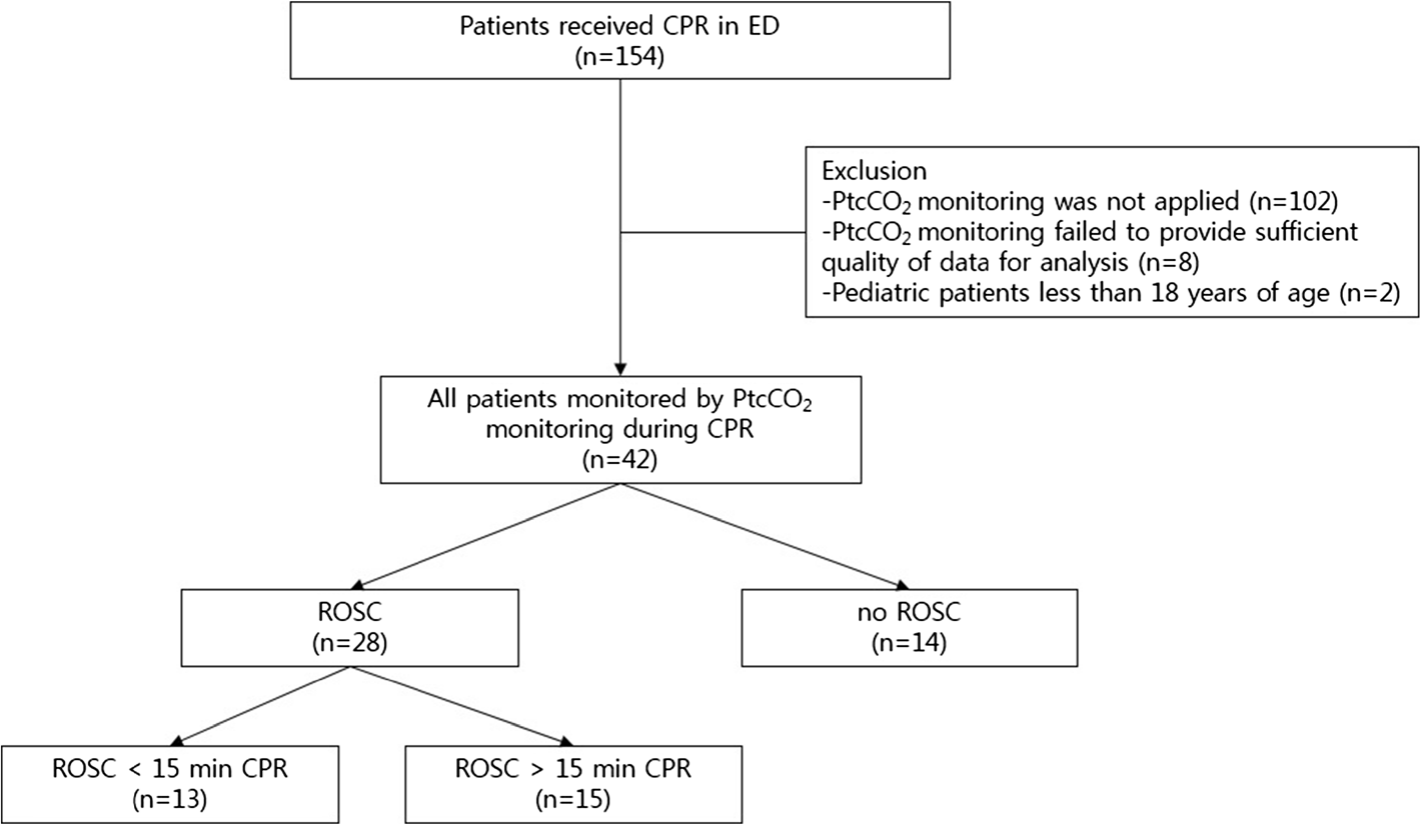
**Patient flow chart.** CPR, cardiopulmonary resuscitation; ED, emergency department; PtcCO_2_, partial pressure of transcutaneous carbon dioxide tension; ROSC, return of spontaneous circulation.

**Figure 2:**
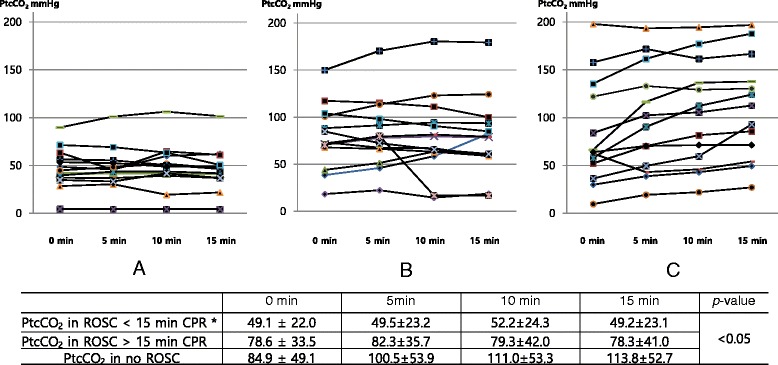
**The absolute value of PtcCO**_**2**_ **over time in patients with cardiopulmonary resuscitation.** PtcCO_2_, partial pressure of transcutaneous carbon dioxide tension; ROSC, return of spontaneous circulation; CPR, cardiopulmonary resuscitation. *p <0.05 vs. PtcCO_2_ in no ROSC according to a post hoc test (**A**. ROSC < 15 min CPR, **B**. ROSC > 15 min CPR, **C**. No ROSC).

**Figure 3:**
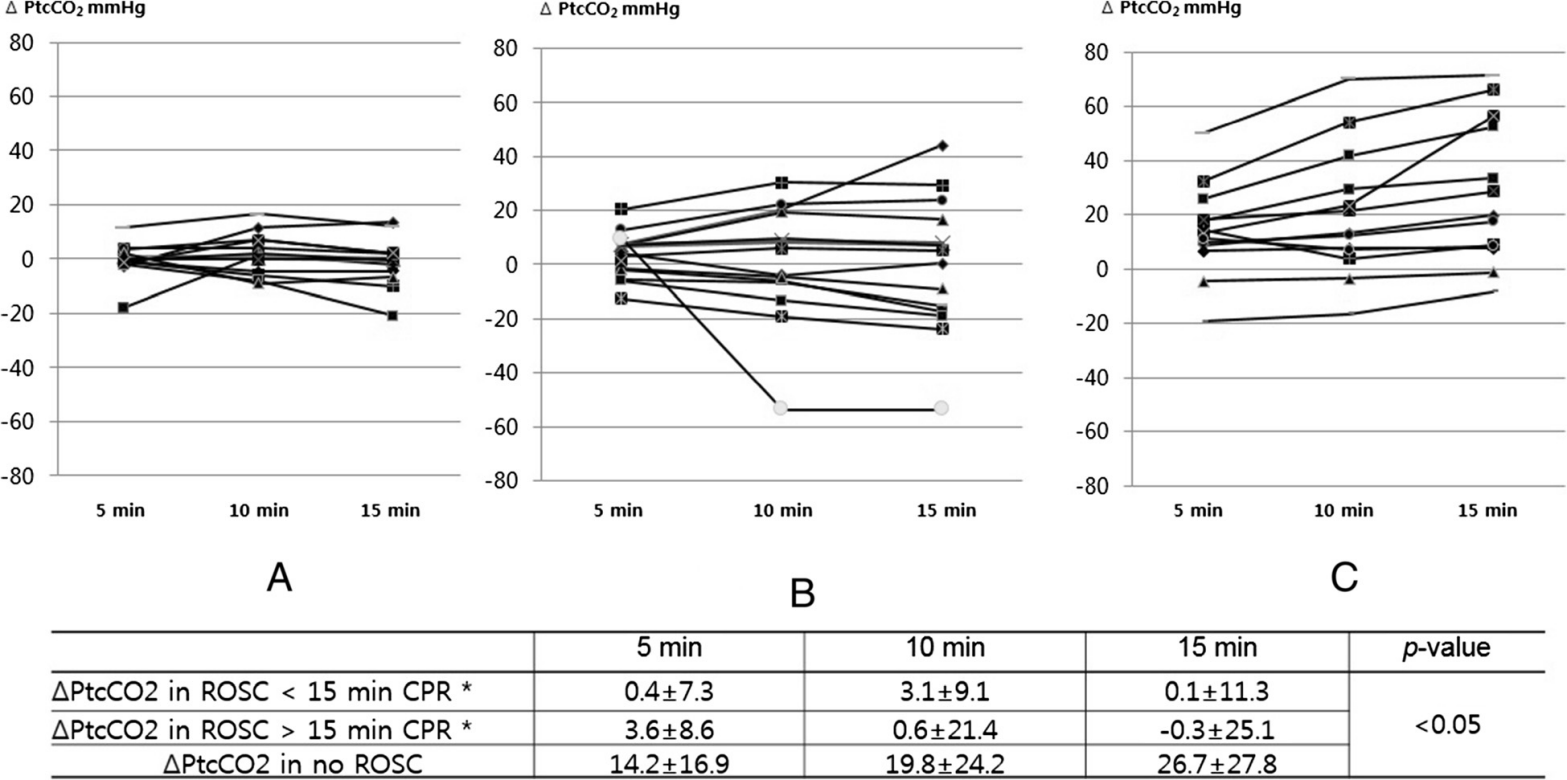
**The change in PtcCO**_**2**_ **over time in patients with cardiopulmonary resuscitation.** PtcCO_2_, partial pressure of transcutaneous carbon dioxide tension; ROSC, return of spontaneous circulation; **△**PtcCO2, the change in PtcCO_2_ from baseline. *p <0.05 vs. PtcCO_2_ in no ROSC according to a post hoc test (**A**. ROSC < 15 min CPR, **B**. ROSC > 15 min CPR, **C**. No ROSC).

A total of 154 patients received CPR in the ED during a 10-month period. One hundred twelve patients were excluded because PtcCO_2_ monitoring was not applied to them; PtcCO_2_ monitoring failed to provide sufficient quality of data for the analysis; or they were pediatric patients under the age of 18 years. In the end, 42 patients were enrolled (Figure 1). Clinical data on the included patients are presented in Table 1. Thirteen patients achieved ROSC in less than 15 min from the start of CPR, 15 patients achieved ROSC after 15 min CPR, and 14 patients expired without ROSC. Age, gender, bystander CPR and OHCA versus IHCA did not show differences among the three groups. Time from EMS call to hospital arrival in OHCA was 25.3 ± 10.4 min in the ROSC <15 min CPR group, 31.4 ± 6.5 min in the ROSC >15 min CPR group and 27.8 ± 14.6 min in the no ROSC (*p* >0.05). The rate of sodium bicarbonate use was the highest in the ROSC >15 min CPR group, and the duration of CPR in the hospital was the longest in the no ROSC group. Initial PtcCO2 was 49.1 ± 22.0 mmHg in the ROSC <15 min CPR group, 78.6 ± 33.5 mmHg in the ROSC >15 min CPR group and 84.9 ± 49.1 mmHg in the no ROSC group (*p* <0.05). The absolute values of PtcCO_2_ were higher and more widely distributed widely in the no ROSC patients than in the ROSC patients (Figure 2). The change in PtcCO_2_ over time, relative to baseline, is shown in Figure 3. The PtcCO_2_ change over time had a tendency to decrease or to remain constant in the ROSC patients. In contrast, all patients in the no ROSC group experienced an increase in the PtcCO_2_ change during CPR except in one case.

**Table 1:** **Clinical data of included patients**

	**ROSC**			
	**Before 15 min CPR (n = 13)**	**After 15 min CPR (n =15)**	**No ROSC (n = 14)**	***p*** value*
Baseline characteristics				
Age, years	62.1 ± 13.0	58.1 ± 16.8	56.7 ± 15.7	0.733
Male sex	9(69.2)	10(66.6)	12(85.7)	0.964
Event characteristics				
Bystander CPR	5 (38.5)	8 (53.3)	8 (57.1)	0.593
OHCA vs. IHCA	4:9	7:8	9:5	0.218
Time from EMS call to hospital arrival in OHCA patients, min	25.3 ± 10.4	31.4 ± 6.5	27.8 ± 14.6	0.549
Duration of CPR in hospital, min	7.0 ± 4.1	36.6 ± 16.6	56.5 ± 26.1	<0.001
Epinephrine used in the first 15 min of in-hospital CPR, mg	2.1 ± 1.0	4.5 ± 1.9	3.9 ± 1.0	<0.001
Sodium bicarbonate use	5 (38.5)	14 (93.3)	5 (35.7)	0.002
Initial PtcCO_2_	49.1 ± 22.0	78.6 ± 33.5	84.9 ± 49.1	0.030

Continuous variables are represented as mean ± standard deviation. Categorical variables are represented as count (%). ROSC, return of spontaneous circulation; CPR, cardiopulmonary resuscitation; OHCA, out-of-hospital cardiac arrest; PtcCO_2,_ partial pressure of transcutaneous carbon dioxide. *represents statistical significance among the three groups.

## Discussion

The chance of survival from cardiac arrest cannot be accurately predicted by physicians. Decisions concerning CPR are difficult in two ways. First, it would be better to stop CPR in cases such as terminal cancer or prolonged CPR. Second, however, if ROSC does not occur, extending the resuscitative effort may be appropriate in some cases [13]. Nowadays, the increasing role of extracorporeal cardiopulmonary resuscitation (ECPR) is highlighted for patients failing conventional resuscitation [14]. Despite the fact that opinions on the indications for ECPR vary among operators, ECPR was provided to patients with witnessed cardiac arrest and a brief “no flow” time; if there was no recovery of cardiac function within 20 min of CPR [15,16]. Thus, ROSC has a large significance in the determination of continuing CPR, while it is also meaningful for planning ECPR in a case with a low possibility of ROSC.

In the case of cardiac arrest, with the assumption that the CO_2_ production is constant, CO_2_ accumulates within the blood in the arrest patient as it cannot be washed out through ventilation. If systemic blood and pulmonary blood flow are recovered by CPR, the PCO_2_ value will drop while increasing the chance of ROSC. However, continuous gas monitoring is difficult during CPR. Invasive monitoring such as arterial blood gas analysis or central ScvO_2_ can be performed after patient stabilization. Several studies confirmed that the etCO_2_ is correlated with the cardiac output produced by CPR. In recent years, etCO_2_ monitoring by capnography has been recognized as a useful procedure in monitoring cardiac arrest patients [17-19]. It has the advantage of easy application and non-invasiveness. Nevertheless, it has some limitations in a CPR situation as noted in the introduction.

As shown in Figure 2, the absolute values of PtcCO_2_ were higher and more widely distributed in the no ROSC patients than in the ROSC patients; PtcCO_2_ becomes flow-dependent during a low-flow shock [11,12]. Figure 3 represents the change in PtcCO_2_ in each patient during CPR. In the no ROSC patients, the PtcCO_2_ value was high and its change increased over time except in one patient. In contrast, these findings were not observed in the ROSC patients. We presumed that the decreased systemic blood flow with the decreased wash-out of CO_2_ from the lung contributes to these results in the no ROSC group, which indicates that PtcCO_2_ reflects changes in the systemic blood flow and the pulmonary blood flow. While end-tidal CO_2_ monitoring provides an indirect inference for the systemic blood flow change, PtcCO_2_ monitoring has the merit of allowing for the direct assessment of the systemic blood flow change.

As shown in Table 1, clinical characteristics were not different among three groups except for the frequency of sodium bicarbonate use, epinephrine used in the first 15 min in-hospital, and the duration of CPR in the hospital. The difference in epinephrine used in the first 15 min in-hospital and the duration of CPR in the hospital were due to dividing the groups by duration criteria. The higher rate of sodium bicarbonate use in the ROSC >15 min CPR group than in the ROSC <15 min may be due to the relatively more unstable clinical condition of the ROSC >15 min CPR group. The higher rate of sodium bicarbonate use in the ROSC >15 min CPR group than in the no ROSC group may be due to the increased success rate of arterial puncture upon recovering the pulse pressure after ROSC. The physicians might have decided to use sodium bicarbonate after confirming acidosis by an arterial blood gas analysis. Therefore, it is likely that sodium bicarbonate was used in a fair number of cases in the ROSC >15 min CPR group after CPR had been administered for at least 15 min. Even if sodium bicarbonate was administrated during the first 15 min of CPR in the ROSC >15 min CPR group, the difference in the PtcCO2 change between the ROSC >15 min CPR group and the no ROSC group would have been much greater.

As shown in Table 1, the duration of CPR was understandably longer in the no ROSC group than in the ROSC >15 min CPR group. The results of PtcCO_2_ in the no ROSC group and in the ROSC >15 min CPR group were not influenced by the CPR time because the analysis of the PtcCO2 change was done within the first 20 min of CPR.

This study has another limitation. Our observations were limited to a small sample. This study was done by retrospective chart review and there was no intervention for applying PtcCO_2_ monitoring during study period. As a result, only 33.8% of the cardiac arrest patients underwent PtcCO_2_ monitoring. Therefore, although this study result seems plausible, further research should be done to confirm this result.

## Conclusion

PtcCO_2_ monitoring provides non-invasive, continuous, and useful monitoring in cardiac arrest patients. Further study is warranted to confirm the use of PtcCO_2_ monitoring for ROSC.

### Provenance and peer review

Not commissioned; externally peer reviewed.
